# DNA Methylation in Ph-Negative Myeloproliferative Neoplasms: Prognostic Role and Therapeutic Implications

**DOI:** 10.3390/cimb47040227

**Published:** 2025-03-26

**Authors:** Paola Barone, Adele Bottaro, Rossana Leanza, Fabio Stagno, Alessandro Allegra

**Affiliations:** Hematology Unit, Department of Human Pathology in Adulthood and Childhood “Gaetano Barresi”, University of Messina, Via Consolare Valeria, 98125 Messina, Italy; paola.barone@polime.it (P.B.); adele.bottaro@polime.it (A.B.); rossana.leanza@polime.it (R.L.); stagnof@unime.it (F.S.)

**Keywords:** epigenomics, DNA methylation, myeloproliferative neoplasms, polycythemia vera, essential thrombocythemia, primary myelofibrosis, methylome, prognosis, oncogenes

## Abstract

Myeloproliferative neoplasms are clonal hematological neoplasms characterized by excessive proliferation of cells of erythroid, granulocytic, and megakaryocytic lineage. The genetic mechanisms underlying this group of blood diseases are now known, but new perspectives have recently emerged in the field of epigenetics and particularly related to the possible role of DNA methylation in disease development and progression. DNA methylation regulates different cellular processes, such as proliferation, differentiation, and apoptosis. In myeloproliferative neoplasms, a link has been found between abnormal methylation patterns, such as hypermethylation of tumor suppressors or, conversely, oncogenes hypomethylation, with the progression of the disease, spreading important prognostic and therapeutic implications. This review aims to investigate the relationship between methylation alterations and myeloproliferative neoplasms, emphasizing the ways by which epigenetic dysregulation promotes disease biology.

## 1. Introduction

Polycythemia vera (PV), essential thrombocythemia (ET), and primary myelofibrosis (PMF) are neoplastic diseases known as Philadelphia-negative (Ph−) chronic myeloproliferative neoplasms (MPNs) [1]. Their main feature is represented by an increased proliferation of blood cell lines, accompanied by bone marrow fibrosis in different grades, especially in PMF [2,3,4,5,6]. On the genetic profile, in 2005, the first mutation was discovered: *JAK2V617F*. It is a gain-of-function mutation causing cytokine independent proliferation of the hematopoietic progenitor cells constitutively activating downstream pathways such as JAK-STAT [7,8,9]. In the following years, two other mutations, calreticulin (*CALR*) and thrombopoietin receptor (*MPL*), were discovered. They are considered “driver mutations” because of their role in determining the MPN phenotype and being major criteria for diagnosis.

JAK2V617F activates erythropoietin (EPO), thrombopoietin (TPO), and granulocyte-colony stimulating factor (G-CSF) receptors in the absence of ligands, whereas exon-12 mutations activate the EPO receptor, resulting in erythroid-dominant myeloproliferation and reduced leukocytosis and thrombocytosis [10]. A crucial element of the disease is the allele burden: an elevated mutant allele burden (>50%) is linked to an increased risk of thrombosis and fibrotic progression [11].

The second detected mutation is CALR, a 46-kDa chaperone protein found in the endoplasmic reticulum (ER) lumen [12]. There are two types of CALR mutations: type-1 and type-2 [13]. Type-1 mutations are associated with a significantly higher risk of myelofibrotic transformation. The role of mutant CALR is not well understood. In recent years, it has been proven that CALR binds to MPL in the ER, constitutively activating it [14]. The third discovered mutation is MPL, which serves as the cell surface receptor for TPO. W515L and W515K are the most common types [15]. The clinical and histological appearance of MPN without the expression of any of these mutations is known as “triple negative” (TN) [16].

Patients with these mutations may show additional mutations, either germline or somatic [17,18]. Despite the obvious link between MPNs and the JAK-STAT point mutation, numerous other molecular processes have been found to be relevant to the pathophysiology of MPNs. The MPN phenotype brought on by the JAK2V617F mutation is most likely modified by other inherited or acquired genetic modifiers in addition to JAK2 [19]. Furthermore, clonal analysis has shown JAK2 wild-type patients with EPO-independent erythroid colony formation. Thus, it is evident that there must be other aberrations causing proliferation and that cytogenetically abnormal clones with or without the JAK2V617F mutation can be identified [20]. DNA methylation, a major method of epigenetic control, includes the insertion of a methyl group to cytosine residues in CpG dinucleotides, resulting in transcriptional repression or activation depending on the genomic context [21].

Through our review, we tried to investigate the role of epigenetics and DNA methylation in the pathogenesis and progression of MPNs. It is known that aberrant DNA methylation patterns, such as hypermethylation of tumor suppressor genes or hypomethylation of oncogenes, are common in MPNs, and they have been linked to deregulation of key cellular processes such as proliferation, differentiation, and apoptosis [22]. We also displayed the implications that these notions have on therapeutic choice and on the influence that therapies can have on the methylome itself, which could become a new fundamental element in the patient’s clinical assessment.

### 1.1. Epigenetic Modifiers

It is known that chromatin is made up of DNA and histones [23], which can undergo a variety of changes, including phosphorylation, methylation, and acetylation. Regarding histones, methylation can modify both DNA and RNA, hence controlling gene expression [24,25]. Cancer cells frequently exhibit epigenetic abnormalities, which manifest as DNA methylation, including genome-wide hypomethylation and site-specific hypermethylation [26]. The genesis of cancer is heavily influenced by genomic instability. Epigenetic changes, such as reversible chromatin shape, histone modifications, and DNA methylation, determine whether genes are activated or silenced [27]. Disruptions in chromatin organization, histone modification, DNA methylation, or RNA processing can cause alterations in gene expression, thereby affecting the initiation, development, or persistence of malignant cells. In nature, these processes are reversible and strive to control proliferation, cell survival, and terminal differentiation [28,29,30].

### 1.2. Methylation Markers

Though few are used for clinical diagnosis, a number of methylation markers have been found to date that are related to a wide spectrum of malignancies, including gastric cancer, hepatocellular carcinoma, lung cancer, colorectal cancer, and esophageal cancer. However, recent research shows that advances in early tumor detection, diagnosis, and whole-course tumor care are gradually expanding the potential for DNA methylation markers in “in vitro” diagnostics and precision medicine. Additionally, RNA methylation modifications (of which m6A RNA methylation is the most prevalent and abundant post-transcriptional modification in eukaryotes) such as 6-methyladenosine, 5-methylcytosine, and 1-methyladenosine are also strongly linked to carcinogenesis [31].

Several cancer-specific genes, including *SOX17*, *TAC1*, *HOXA7*, *CDO1*, *HOXA9*, and *ZFP42*, have been found to exhibit high-frequency methylation profiles in plasma and sputum samples from patients with lymph node-negative stage I and IIA non-small cell lung cancer [32]. In addition, it has been shown that the putative universal cancer-only methylation marker HIST1H4F was hypermethylated in a number of cancers, including lung cancer, indicating a general role in carcinogenesis and being a potential target for early detection and screening [33].

However, the first and most widely studied DNA alteration is 5-methylcytosine (5-mC). DNA methyltransferases (DNMT1, DNMT3A, and DNMT3B) perform this modification, whereas ten-eleven translocase (TET) enzymes—TET1, TET2, and TET3—catalyze the conversion of 5-mC to 5-hydroxymethylcytosine (5-hmC), which requires the cofactor alpha-ketoglutarate (AKG) produced by isocitrate dehydrogenase (IDH1 and IDH2) enzymes [34]. DNMT1 acts as a methyltransferase that maintains methylation patterns during cell division. It may also methylate previously unmethylated DNA. DNMT3A and DNMT3B are de novo methyltransferases that specifically target unmethylated cytosines. TET enzymes oxidize 5-mC to 5-hmC in the presence of AKG, a metabolite produced by IDH1/2 activity. TET2 and TET3 are largely expressed in hematopoietic cells, whereas TET1 is predominantly found in embryonic stem cells [35]. Histone modifications also play an important role in determining cell function, resulting in different states of chromatin structure. Histone acetyltransferases (HATs) are required for chromatin relaxation, whereas histone deacetylases (HDACs) are active later in erythropoiesis, contributing to transcriptional silencing. Histone methyltransferases, on the other hand, are particularly active during the proliferative stages of erythroid development [36,37,38,39,40]. Aside from the primary driver mutations, MPNs typically have mutations in genes involved in epigenetic control and mRNA splicing. The most common pathogenic mutations are found in the above-mentioned epigenetic regulators TET2 and DNMT3A, as well as in EZH2. This epigenetic modulator Enhancer of Zeste homolog 2 is a catalytic subunit of Polycomb Repressive Complex 2 (PRC2), which methylates histone H3 lysine 27 (H3K27me3), resulting in transcriptional repression [41].

In MPNs, EZH2 loss-of-function mutations activate oncogenes and enhance hematopoietic stem cell (HSC) self-renewal. Other key genes involved in pathogenesis include *ASXL1* and Ikaros (*IKZF1*). ASXL1 increases the activity of both the PRC1 and PRC2 complexes. Mutations in *ASXL1* diminish H3K27 methylation and alter PRC2 function, notably the recruitment of EZH2. IKZF1 represses myelopoiesis genes via interactions with histone deacetylase complexes. IKZF1 deletions are present in 21% of blast-phase MPN patients but just 0.2% of chronic-phase cases, indicating a key role in leukemic transition. These genetic alterations contribute to MPNs’ complicated pathophysiology by influencing differentiation, self-renewal, and gene expression via epigenetic and transcriptional dysregulation [42]. Finally, gain-of-function mutations in the IDH1 and IDH2 genes have been found in a small number of individuals with myelodysplastic syndromes (MDS) and MPNs [43]. These mutations occurred in 1.9% of PV patients, 0.8% of ET patients, and 4.2% of PMF patients. However, in blast-phase MPN, IDH mutations were found in 21.6% of patients and were related with lower survival rates. IDH1/2 mutations limit the activity of α-ketoglutarate (aKG)-dependent enzymes, such as TET2 and Jumonji C-terminal domain histone demethylases, which are essential for normal DNA and histone methylation [44] (Table 1).

### 1.3. JAK2 as a Methylome Agent

JAK2 signaling plays a crucial role in different cellular processes, such as cell cycle progression, genetic stability, apoptosis, and chromatin remodeling [45]. In MPNs, mutant JAK2 proteins gain novel functions, including altered interactions with the enzyme to protein arginine methyltransferase (PRMT5). Mutant JAK2 binds more strongly PRMT5 than wild-type JAK2, phosphorylating and impairing PRMT5′s ability to methylate histone proteins. This disruption in histone methylation contributes to the MPN phenotype by modifying chromatin structure and gene expression. Additionally, nuclear localization of JAK2 has been observed, where it phosphorylates histone H3 by displacing HP1α and leading to the overexpression of oncogenes responsible for leukemogenesis [20]. Studies have reported that the activated JAKV617F mutation reduces methylation levels of the polycythemia rubra vera 1 gene (prv-1), which is a GPI-linked protein produced on neutrophils [46]. Under normal circumstances, prv-1 is expressed in a subpopulation of neutrophils, but its mRNA levels are significantly raised in Ph- MPNs, including PV. Moreover, prv-1 mRNA levels are inversely related to the degree of prv-1 gene methylation at the C30 location in PV and ET patients [47]. Recent evidence also suggests that JAK2 signaling modulates the epigenetic regulation of prv-1 gene expression [48]. These findings show that the JAK/STAT system influences gene expression via epigenetic processes, which supports its function in regulating prv-1 gene methylation and expression. One study also linked the JAK2V617F mutation to the breakdown of the SDF-1/CXCR4 axis in PMF patients [49]. SDF-1 and its receptor, C-X-C chemokine receptor type 4 (CXCR4), are important chemoattractants for hematopoietic stem and progenitor cells (HSCs/HPCs) produced by bone marrow microenvironment components such as endothelial cells, immature osteoblasts, stromal cells, and CD34 cells [50]. In PMF, the mobilization of CD34 cells into peripheral blood is a hallmark feature, with their levels exceeding those in healthy individuals by over 300 times and surpassing other myeloproliferative disorders by 20–30 times [51]. An increased number of circulating cells expressing CD34 has been observed, especially in the early disease stages. The mobilization of CD34 cells in PMF is likely influenced by a proteolytic environment within the bone marrow, degrading SDF-1 [52]. Furthermore, CXCR4 abnormalities contribute to this occurrence, as indicated by low CXCR4 expression and mRNA levels in CD34 cells from PMF patients. Additionally, increased plasma levels of neutrophil elastase, MMP-9, and soluble vascular cell adhesion molecule-1 correlate with circulating CD34 cell counts [53].

CXCR4 has been identified as a target for epigenetic regulation in PMF. Reduced CXCR4 expression in CD34 cells from PMF is associated with hypermethylation of its promoter. This points to a protease-independent mechanism for HSC/HPC mobilization. Shi et al. found that chromatin-modifying drugs such as 5-AzaD, a hypomethylating agent, and trichostatin might repair epigenetic changes [42,54]. In long-term cultures, treated CD34 cells produced less clonogenic mutant HPCs with JAK2V617F and more normal CXCR4-expressing cells [55,56].

## 2. Methylome in MF and the Role of microRNA

The evaluation of global methylation profiles reveals that PMF is epigenetically distinct from other MPNs, with PV and ET cases showing significant hypermethylation in genes like the transcription factor *HNF4α*, histone acetyltransferase *MYST2*, and *interleukin-1*. In contrast, PMF exhibits both hypermethylation and hypomethylation, with hypomethylation being more prominent, 49% in PMF as compared to 38% in PV and ET [57]. Hypomethylated genes in PMF are linked to pathways involving cell signaling, hematopoiesis, and immune response, while hypermethylated genes are associated with inflammatory pathways [58]. Mutations in *ASXL1* and *TET2* further exacerbate these epigenetic disruptions, with *ASXL1* mutations increasing hypermethylation and *TET2* mutations leading to both hypermethylation and reduced hydroxymethylation [59]. The predominance of hypomethylation in PMF represents a unique epigenetic pattern that impacts hematopoietic differentiation and neoplastic transformation. In fact, both hypo- and hypermethylated CpG sites are found to be underrepresented in classical CpG islands (CGIs) and predominantly enriched in regions outside these islands. Interestingly, these alterations are concentrated in enhancer regions rather than promoters, a notable deviation from patterns seen in other cancers [60]. Enhancer methylation is increasingly recognized as a critical regulator of gene expression, and its involvement in MF suggests a unique mechanism driving the disease [61].

**Figure 1:**
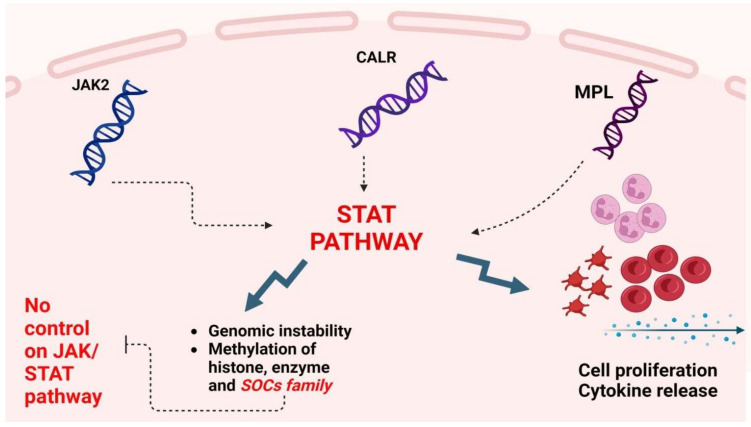
Consequences of JAK2, MPL, and CALR mutations on cell proliferation and genomic instability. Created in https://BioRender.com (accessed on 20 February 2025).

Another actor recognized in this scenario is a zinc-finger protein (ZFP36L1) responsible for degrading unstable mRNA. The hypermethylation of its enhancer region suppresses ZFP36L1 expression, disrupting its tumor-suppressive function [62]. When this hypermethylation is reversed by treatment with azacytidine, cell proliferation is significantly reduced in vitro [63]. Furthermore, SOCS proteins, which are essential regulators of the JAK/STAT pathway, play an important role in MPN pathogenesis [64]. The JAK2V617F mutation is responsible for the formation of abnormal transcriptional profiles, precisely through DNA methylation. Studies focusing on individual loci have identified aberrant promoter methylation in genes such as *Suppressor of Cytokine Signaling 1* and *3* (SOCS-1, SOCS-3) [65,66]. SOCS proteins are among the most thoroughly researched inhibitors of this signaling pathway. This family consists of eight members, including cytokine-inducible SH2 domain protein (CIS) and SOCS-1 through SOCS-7, which are typically expressed at low levels in resting cells [67]. When cells are active, SOCS protein production increases, suppressing activated STATs and controlling cytokine signaling. Notably, acquired abnormalities in these regulatory proteins can cause excessive STAT activation, even in the absence of JAK2 mutations [68]. SOCS proteins have an Src homology-2 (SH2) domain that binds to phosphotyrosine residues on cytokine receptors (e.g., SOCS-2, SOCS-3, CIS) and JAK kinases (e.g., SOCS-1) [69]. Moreover, SOCS proteins reduce JAK activity, compete with STATs for receptor binding sites and induce protein degradation, all of which suppress cytokine signaling. Gene deletion studies have revealed the selectivity of various SOCS proteins [70]. Furthermore, SOCS proteins may operate as tumor suppressors, with hypermethylation of SOCS genes, thus resulting in transcriptional silence and potentially contributing to cancer development [71]. SOCS-1 hypermethylation, for example, has been identified in acute myeloid leukemia and MDS, and patients with ET and PV have similar abnormalities in the SOCS-1 or SOCS-3 genes [72]. Surprisingly, JAK2V617F mutations can escape SOCS-mediated control, as evidenced by studies showing that this mutation suppresses SOCS-3 degradation and causes tyrosine hyperphosphorylation while paradoxically increasing its kinase activity [73]. Other published data suggest that SOCS-1 and SOCS-3 hypermethylation affects gene transcription in MPN patients, since granulocytes from these individuals show considerably lower SOCS expression than healthy controls, even when activated by cytokines [Figure 1].

**Figure 2:**
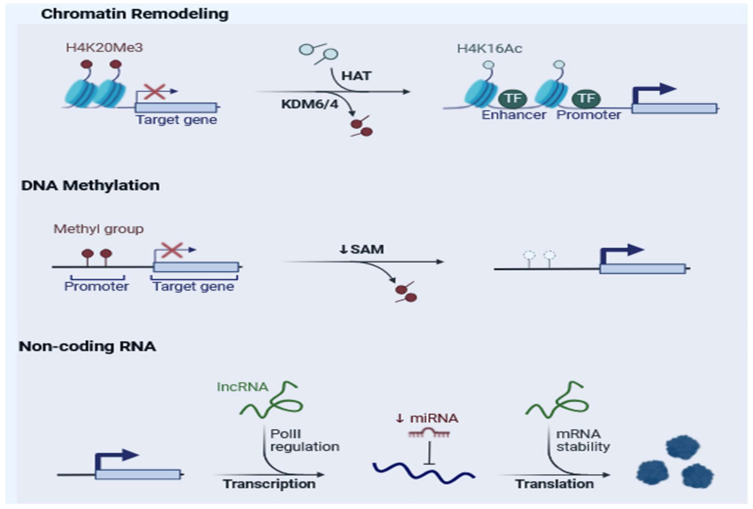
The main biological mechanisms underlying epigenetic mutations. Chromatin remodeling: loss of H4K20me3 and gain of H4K16Ac mark an increased transcription of the target gene. DNA methylation: loss of DNA methylation results in aberrant transcription of target genes. Non-coding RNA: non-coding RNA regulation can alter transcription and translation of oncogenic gene targets. Created in https://BioRender.com (accessed on 20 February 2025).

Recent studies have connected hypermethylation of microRNAs (miRNAs) to cancer development. miRNAs are single-stranded, non-coding RNA molecules made up of 22–25 nucleotides that control protein production by decreasing the amounts of their target proteins [74,75,76,77]. They can act as tumor suppressors or oncogenes. For instance, the epigenetic silencing of miR-34b, a component of the p53 regulatory pathway, has been implicated in AML [78]. Similarly, methylation of the miR-124-1 promoter, the first tumor suppressor miRNA identified as being regulated by DNA methylation, has been associated with poor outcomes in acute lymphoblastic leukemia (ALL). In chronic myeloid leukemia (CML), hypermethylation of miR-203 has been shown to confer a growth advantage to tumor cells by downregulating the oncogenic BCR-ABL fusion protein [79,80]. In MPNs, available information is scarce. The study by Chim CS et al. has shown that there are some miRNAs involved in the process of erythropoiesis and in the differentiation of megakaryocytes, such as miR-451, upregulated in erythropoiesis, and miR-10a, -10b, -17, -20, -106, and -126 and miR-99a, -101, -126, and -135, respectively downregulated and upregulated in the maturation of megakaryocytes. Moreover, it has been shown that the silencing of miRNAs with a role as tumor suppressors, such as miR-34a, -34b/c, and -124-1, favors the proliferation of tumor cells. miR-203 and miR-34b/c methylation was found in 15.6% of patients affected by MPNs. This suggests that miR-203 methylation is a common feature across different MPNs and leukemias, regardless of Philadelphia chromosome status, indicating a broader role in these hematologic malignancies [81,82,83,84] (Figure 2).

Finally, noncoding RNAs greater than 200 base pairs are referred to as long noncoding (lnc) RNAs [85]. Only a small portion of the 15,778 human lncRNAs that have been identified so far [86] have been characterized. They serve as the primary regulators of several cellular processes, including transcriptional and post-transcriptional regulation, nuclear-cytoplasmic transit, cell proliferation, cellular architecture, and cell cycle progression. Additionally, they have an impact on the epigenetic control of gene expression [87].

The clinical significance of rs6983267 and the related CCAT2 lncRNA linked to cancer risk was successfully determined by a study. Both alleles are significant because constitutive overexpression of allele-specific CCAT2 changed the hematopoietic cells’ global gene expression landscape, which in turn caused de novo myeloid malignancies. Additionally, they discovered rs6983267-RE, a novel SNP-associated RNA mutation that is extremely common in cancer cells. This mechanism may allow the aberrant malignant cells to preferentially create heterozygous transcripts from homozygous DNA and regulate the transcription of the rs6983267 SNP locus (as well as maybe a number of additional loci to be found). Actively transcribed SNPs may have a more fundamental role in genomic control than previously thought, according to this study. Thus, rs6983267-RE is a finely controlled event in human MDS/MPN [88].

## 3. Chronic Inflammation and Methylome

The persistent inflammatory state in MPNs positions this group of neoplasms as a potential “Human Inflammation Model” [89,90]. Chronic inflammation is characterized by prolonged immune cell activation, DNA damage, tissue destruction, fibrosis, and tissue remodeling, all of which are exemplified in MPNs, particularly in the myelofibrosis stage [91,92]. Increased DNA methylation is another consequence of chronic inflammation, with levels progressively rising during tumor development [93]. Evidence in MPNs demonstrated abnormal DNA methylation patterns, particularly pronounced in advanced stages like MF, while less evident in ET and PV [94]. These findings suggest that the chronic inflammatory environment in the bone marrow may contribute to epigenetic alterations, genomic instability, and DNA mutations in hematopoietic cells [95]. These changes could not only initiate clonal development but also drive clonal evolution by promoting additional mutations [96,97,98,99,100,101] (Table 2).

An investigation study showed that the beginning of myelofibrosis and advanced myeloproliferative neoplasm phenotypes are linked to ASXL1 mutations. ASXL1 mutations increase inflammation and bone marrow fibrosis, causing skewed monocyte/macrophage and neoplastic monocyte-derived fibrocyte differentiation. In hematopoietic stem and progenitor cells, the deletion of ASXL1 and JAK2V617F mutations consistently results in increased activation of polycomb group target genes, participating in the epigenetic control of gene expression, including EGR1. Increased hematopoietic stem and progenitor cell commitment to the monocyte/macrophage lineage is thereby explained by the activation of EGR1. Additionally, EGR1 stimulates TNFA activation, which promotes monocyte development into fibrocytes. Thus, ruxolitinib and TNFR antagonist therapy dramatically lower fibrocyte formation in vitro [102].

**Figure 3:**
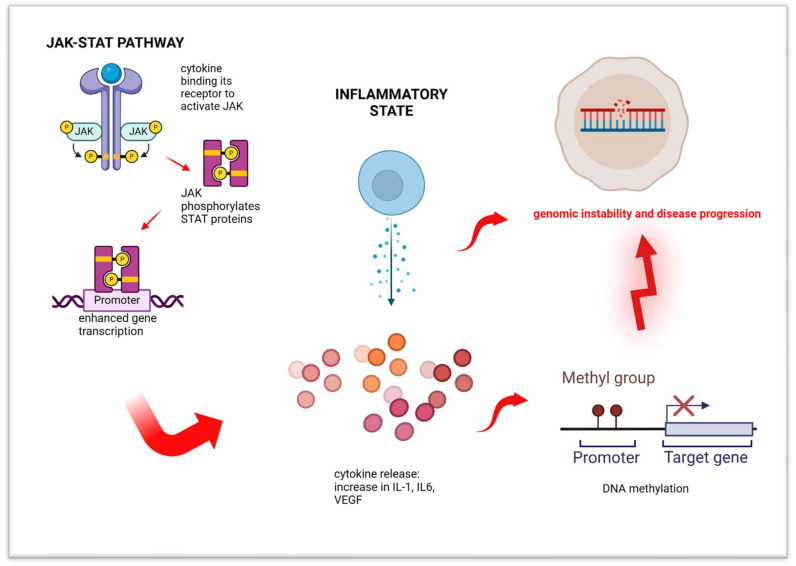
Interaction among JAK2-STAT signaling, inflammation, and DNA methylation. Created in https://BioRender.com (accessed on 20 February 2025).

Many inflammatory cytokines in MPNs are not directly linked to driver mutations such as JAK2V617F, CALR, or MPL. For instance, JAK2V617F has been associated with the production of IL-1β and IP-10, but cytokine production in CALR-mutated cases often originates from non-mutated T-cells. Non-driver mutations, such as those in DNMT3A, TET2, SRSF2, and SF3B1, may indirectly contribute to inflammation by activating the NF-κB pathway, which stimulates key inflammatory cytokines like IL-1β and TNF-α [103,104,105]. Inflammation not only drives myeloproliferation but also promotes genomic instability and aberrant DNA methylation, particularly in MF patients. Pro-inflammatory cytokines such as IL-6 and TNF-α, along with oxidative stress, play a crucial role in these epigenetic alterations (Figure 3). This chronic inflammatory environment fosters clonal evolution by triggering additional mutations, advancing the disease from ET or PV to MF [106]. JAK-STAT activation, together with the JAK2V617F mutation and JAK2 overexpression, contributes to genomic instability and increased mutagenesis. This highlights the potential of targeting inflammation as a therapeutic strategy. Early intervention with anti-inflammatory therapies could prevent inflammation-driven mutagenesis and disease progression. JAK1/2 inhibitors, such as ruxolitinib, are effective in reducing inflammatory cytokines and inhibiting mutant clones, potentially slowing the progression to MF [107]. Additionally, IFN-alpha2 has shown promise in reducing the JAK2V617F mutation burden, enhancing tumor immune surveillance, and targeting mutant clones at the stem cell level [108]. Combining JAK1/2 inhibitors with IFN-alpha2 represents a compelling therapeutic strategy, as it addresses inflammation, reduces mutational load, and enhances immune function. Moreover, targeting the epigenome with hypomethylating agents or histone deacetylase inhibitors (HDACi) may further mitigate chronic inflammation and reduce the risk of additional mutations, ultimately slowing disease progression [99].

## 4. Apoptosis and Epigenomics in MPNs

Several studies have analyzed not only the proliferation processes but also those of cell death. It has also been highlighted that epigenetic mechanisms of DNA methylation underlie apoptosis. This link is evidenced by the genetic pathogenesis of MPNs. In fact, JAK2, MPL, and CALR mutations have a role in megakaryocytes survival [109]. 

Neoplastic megakaryocytes show higher levels of the anti-apoptotic protein Bcl-XL. Concurrently, there is a significant reduction in the pro-apoptotic protein BNIP-3, limiting megakaryocytes’ ability to oppose Bcl-XL survival signals and suppressing apoptosis [110,111].

This decrease in BNIP-3 is notably prominent in cases of MF, implying a reduced pro-apoptotic potential when MPNs shift to the “accelerated” phase (particularly in CALR-mutated patients) [112]. Morphological investigations also show that tiny megakaryocytes with compacted nuclear chromatin (characteristic of MF) are negative for BNIP-3. These changes, which indicate non-necrotic cell death or “para-apoptosis”, could be the result of Bcl-XL resistance or decreased calreticulin function [113]. In CALR-mutated cases, aberrant calreticulin signaling might compromise appropriate BNIP-3 folding, resulting in its degradation and/or failed caspase-8 activation, hence hindering megakaryocyte death. This evidence supports a pathophysiological profile in which megakaryocyte survival in MPNs is determined by proliferative and apoptotic mechanisms controlled by upstream JAK-STAT and calreticulin signaling pathways. Interestingly, MPNs carrying the JAK2V617F mutation have higher BNIP-3 expression than “double-negative”. Once the JAK2V617F mutation is acquired, megakaryocytes may initiate an intrinsic apoptotic cascade in response to severe, irreversible DNA damage [114]. The previously lowered BNIP-3 levels may thereafter partially rise, facilitating megakaryocyte death. Alternatively, enhanced BNIP-3 could be a compensatory response by megakaryocytes to offset the anti-apoptotic effects of high Bcl-XL [115]. Notably, ET cases show a slightly less pronounced downregulation of BNIP-3 compared to PMF [116,117].

## 5. The Complexity of Modern Epigenetic Methods in Biological Research

In recent years, the field of epigenetics has had exponential growth, driving the need to develop new analysis techniques of capable of accurately deciphering epigenetic modifications and their impact on both gene regulation and proteins involved in cellular processes. Epigenetic modifications, such as DNA methylation, histone modifications, and the action of non-coding RNAs, profoundly influence gene expression without altering the DNA sequence. This makes the use of advanced methodologies for their identification and characterization indispensable. The use of sophisticated technologies allows us to identify hidden patterns and complex correlations between epigenetic modifications and the development of diseases.

The gold standard method for detecting DNA methylation is whole genome bisulfite sequencing (WGBS), an inventive technique for mapping methylation profiles at single base resolution. Through this method, methylated cytosines in genomic DNA are unharmed by bisulfite treatment, whereas unmethylated cytosines are transformed into uracils. Whole genome sequencing is then used to differentiate between methylation sites and levels throughout the genome [118]. Because of its high-throughput capabilities and broad detection range, WGBS has begun using methylation mapping in clinical diagnostic applications for human disorders. For example, WGBS has been used to determine hydroxymethylation profiles for cervical cancer progression stages in order to screen for possible epigenomic biomarkers [119] or to identify differentially methylated regions (Dlgap1, TMEM51, and Eif2ak2) in the brain tissues of Alzheimer’s disease model mice [120]. Methylome analysis in human alveolar and bronchial epithelial tissue was obtained more recently using WGBS [121]. Although WGBS offers many benefits, including a wide coverage of methylation sites and less interference from repetitive sections, SNPs, and other variables [122], it also has disadvantages, such as low alignment rates and poor sequence alignment precision.

A different established technique for evaluating protein–chromatin interactions at known binding sites is chromatin immunoprecipitation-quantitative real time PCR (ChIP-qPCR). This method measures occupancy at promoter regions and assesses modification status by using antibodies to concentrate DNA segments with histone modifications [123]. For instance, by identifying H3K18la enrichment at the YTHDF2 promoter, ChIP-qPCR was recently utilized to investigate the function of histone lactylation in the metabolic regulation of gene expression. This investigation demonstrated that the H3K18la change upregulates the oncogene YTHDF2 [124]. Chromatin immunoprecipitation sequencing (ChIP seq) is a useful technique for methylation profiling in combination with ChIP-qPCR because it can map DNA-binding proteins and histone modifications throughout the genome at a comparatively high resolution. H3K79 methylation was found to play a role in controlling cisplatin resistance in ovarian cancer through C/EBPβ and DOT1L, according to ChIP seq analysis of the H3K4, H3K36, and H3K79 methylation sites [125].

Furthermore, today we discuss multi-omics, a combination of epigenomic, transcriptomic, and proteomic data to obtain a more complete view of gene regulation and its impact on cellular function. The aim is to discover new epigenetic markers both for the development of rapid and early diagnostic techniques and to identify new therapeutic targets and expand our knowledge on the prognostic aspects of pathologies [126]. Myeloproliferative neoplasms represent a diverse group of tumors: once the subtype is identified, we may ensure direct treatment. However, characterization of the molecular events of MPN is challenging due to the conventional subtype identification techniques that employ a single-omics approach. The limitations of single-omics data can be efficiently compensated for by the identification of subtypes using multi-omics association techniques. An integrated data collection could be produced by combining transcriptomic, proteomic, and epigenomic data. Researchers will also incorporate the relationships between proteins and genes as well as between methylation and genes using this strategy, in addition to integrating multiple omics datasets. Thus, we will be able to effectively supplement multi-omics data.

The starting point for most of the studies was mononuclear cells, granulocytes, or lymphocytes. Samples were extracted from bone marrow or peripheral blood, both from patients and control subjects such as healthy donors [127]. Information about the mutational status was collected retrospectively. DNA methylation studies were conducted using a methylation 450K array kit [128,129], and the data obtained were subsequently analyzed using an open-source software, as reported in the study by Martínez-Calle et al. [76]. In other studies, genomic DNA was extracted from cells after Ficoll density gradient separation and subsequently analyzed with the HELP assay (HpaII tiny fragment enriched by LM-PCR) [130]. Selective digestion of DNA with the enzymes HpaII and MspI, followed by PCR amplification and microarray hybridization, allows large-scale identification of genomic methylation. To confirm the microarray results, quantitative methylation analysis was performed using MALDI-TOF mass spectrometry with the EpiTyper MassArray system [131,132,133]. Bioinformatic analyses were conducted to identify specifically methylated genes and molecular pathways, and the effects of mutations were examined through SNP karyotyping and hydroxymethylation quantification [75]. To correlate a certain degree of methylation of genes such as SOCS with the dysregulation of the JAK/STAT pathway or to study the methylation status of the promoters of specific genes like CXCR4 in CD34+ cells, DNA extraction through bisulfite treatment was performed to analyze the methylation levels of CpG islands in the promoters [131]. A methylation-specific PCR (MS-PCR) technique was employed, and to evaluate the functional effect of methylation, mRNA levels were measured using real-Time PCR. The analysis of SOCS-1 and SOCS-3 proteins was conducted through western blot, both under basal conditions and after stimulation with G-CSF [77]. CD34+ cells were isolated from peripheral blood or bone marrow using density gradient separation techniques and immunomagnetic selection, and their purity was verified through flow cytometry [55]. Additionally, the effect of a demethylating agent, 5-AzaD, was evaluated to explore the potential restoration of gene expression. The expression of CXCR4 was measured using quantitative real-time PCR and flow cytometry [134]. Finally, to investigate the role of the JAK2V617F mutation in inducing genetic instability, murine cell lines (Ba/F3) and CD34+ cells purified from patients with PV and PMF, all positive for the JAK2V617F mutation, were used. The cells were amplified in vitro in the presence of a combination of cytokines (SCF, IL-3, FLT3-L, IL-6, and TPO) to promote proliferation and subsequently analyzed. To evaluate homologous recombination, a fluorescent substrate (HR-EGFP/3′EGFP) was used, enabling the measurement of recombination events through flow cytometry [135]. Alterations were studied by infecting the cells with retroviral plasmids containing either wild-type JAK2 (JAK2wt) or mutated JAK2V617F. Signs of genetic instability included centrosome abnormalities, aneuploidy, and increased sister chromatids exchange. To identify point mutations, deletions, and insertions, spontaneous mutagenesis assays were performed on two gene loci (HPRT and Na/K-ATPase). Additionally, cell resistance to genotoxic drugs such as mitomycin C and bleomycin was used as an indicator of alterations in DNA repair mechanisms [136].

## 6. Present and Future of MPNs Therapy

MPNs treatment has been revolutionized by the discovery of JAK inhibitors (JAKi). From the first and still used hydroxycarbamide (HU, also called hydroxyurea), we now have increasingly targeted and selective therapies with an important influence on the mutational profile. 

### 6.1. Drugs and Their Epigenetic Role

Hydroxycarbamide (HU) is the most well-known and widely used cytoreductive drug in the history of blood neoplasms [137]. As a non-alkylating antiproliferative agent, it is used not only in blood oncology but also in the treatment of conditions such as sickle cell anemia and, in some trials, Alzheimer’s disease. Global DNA hypermethylation has been linked to HU exposure in a number of studies [138,139,140,141]. A single point mutation in the gene for the β-globin subunit of adult hemoglobin (HbA) causes sickle cell disease (SCD), a crippling hereditary condition that affects red blood cells. One research study postulated that certain effects of HU might be mediated by epigenetic modifications, notably DNA methylation, since these changes are critical for erythropoiesis and HbF production [142].

Its antineoplastic action derives from its ability to inhibit DNA synthesis, making its cytoreductive effect particularly useful in myeloproliferative disorders, such as MPNs, CML, and hypereosinophilic syndrome (HES) [143]. HU remains the first-line treatment for high-risk PV and ET patients. It is efficient in lowering leukocyte and platelet counts in people with PV and ET. However, it requires constant administration to ensure continuous suppression of erythropoiesis. Furthermore, it has a variable effect on systemic disease symptoms, such as pruritus and splenomegaly, although no studies have demonstrated the drug’s actual efficacy in prolonging survival. Nevertheless, it remains the first-line drug in the management of MPNs. Its primary mechanism of action is based on the inhibition of enzymes that catalyze the reduction of ribonucleotide diphosphates into their corresponding deoxyribonucleotide triphosphates (dNTPs), which are essential for DNA synthesis and repair. This inhibition leads to depletion of dNTP, resulting in cell cycle arrest. Several alternative mechanisms have been proposed, including the HU effect on DNA methylation, which subsequently influences transcription factors. However, even though it has been used for over 30 years, knowledge regarding the HU role in the methylation process remains limited [144]. Among the HU investigated molecular mechanisms, there is an impact on the expression of hematopoietic regulatory genes, such as *SPI1* and *RUNX1*, which are pivotal for the maintenance and regulation of HSCs. In murine models with the JAK2V617F mutation, HU partially restored the expression of these genes. *SPI1*, an essential regulator for hematopoietic differentiation, is particularly sensitive to the HU action. Regarding its role in DNA methylation, it was observed that HU induces significant changes in DNA methylation in MPN patients, particularly in CD34+ stem cells [145]. Notably, the distal regulatory regions of *SPI1*, which are hypermethylated in patients, ae affected. After HU treatment, promoter methylation is reduced, leading to an increase in *SPI1* expression. A significant difference has also been noted between different cell types: the effect of HU on DNA methylation in mature and differentiated cells, such as neutrophils, was minimal, whereas in CD34+ cells, HU showed a marked response in terms of methylation and gene expression [146,147]. The HU role also extends to molecular pathways regulating the immune system, with particular emphasis on genes involved in inflammation and immunity, suggesting that its action spans multiple biological levels beyond cellular proliferation [22]. Ruxolitinib, the first JAK1/2 inhibitor licensed for the treatment of myelofibrosis, was shown to reduce spleen size and alleviate constitutional symptoms. Long-term follow-up studies indicate that it may also lower allele burden over time. The RESPONSE study found a gradual decrease in the JAK2 allele load in individuals treated with ruxolitinib, establishing the groundwork for using allele burden reduction as an indicator of response to therapy. Interestingly, a recent study found that patients with myelofibrosis who had a greater allele burden (>50%) at the start of treatment did better in terms of spleen size reduction [148,149].

However, ruxolitinib shows a significant limitation: it targets the kinase domains of JAK1/2, with no preference for mutant JAK2. A Mayo Clinic study emphasized the short-term efficacy of ruxolitinib but noted that 92% of patients discontinued treatment by a median of 9.2 months, primarily due to a loss of therapeutic response. Following discontinuation, clonal evolution was observed in 35% of patients and was characterized by the acquisition of new mutations. The most frequently acquired mutations occurred in *ASXL1*, followed by *TET2*, *EZH2*, and *TP53* [150]. Studies on ruxolitinib provide valuable insights into its action in the epigenetic field, highlighting how this molecule impacts both DNA methylation changes and other levels of epigenetic regulation, such as histone modifications [151]. To demonstrate the epigenetic effect of ruxolitinib, Greenfield et al. [152] focused on the histone modification in MPNs cell line models. H3K9 is one of the most extensively studied histones. To investigate whether ruxolitinib treatment alters histone H3 in MPN cell lines, the cell line was treated with 100 nM of ruxolitinib. Western blot examination was then performed with antibodies specific to the methylated form of H3K9. Ruxolitinib-treated cells showed an increase in methylation markers. These data show that ruxolitinib therapy can cause histone changes in MPN cell lines, most likely through the reduction of the JAK/STAT transcriptional drive [149]. In addition to histone modifications, ruxolitinib has been shown to influence DNA methylation (DNAm) patterns. Using an aging signature derived from DNAm profiles of key genes, studies have revealed that PV patients exhibit an epigenetic age older than their chronological one, while ET patients show a younger epigenetic age. Remarkably, ruxolitinib therapy shifted the DNAm age in both groups closer to their actual chronological age, further supporting its epigenetic impact [152,153,154]. Fedratinib is a second-line JAK inhibitor that targets both wild-type JAK2 and the mutant JAK2 V617F, competing for ATP binding. This mechanism effectively inhibits JAK2 activation and suppresses the overactive JAK-STAT signaling pathway. Additionally, fedratinib inhibits FMS-like tyrosine kinase 3 (FLT3), thereby reducing cytokine production mediated by B- and T-lymphocytes [155,156]. Interestingly, fedratinib inhibits BRD4, a member of the bromodomain and extra-terminal domain (BET) protein family. BET proteins are essential for cell proliferation, division, and pro-inflammatory signaling. Fedratinib, which inhibits both the JAK/STAT system and BET proteins, was demonstrated in mice models to lower cytokine production and to reverse bone marrow fibrosis [157]. This combined activity against JAK2 and BRD4 is thought to have a substantial role in the drug’s clinical efficacy, even if studies on its epigenetic impact and that of the recent momelotinib are still in progress [158,159]. The newest JAK1/JAK2 inhibitor, momelotinib (CYT387), has been studied for the treatment of intermediate- to high-risk primary and secondary myelofibrosis (MF) (Table 3). Momelotinib reduces hepcidin expression, which has a direct, favorable effect on anemia and restores erythropoiesis, with 70% of transfusion-dependent patients reaching transfusion independence. In addition, 59% of patients had a significant spleen response, and most of them reported relief from constitutional symptoms. However, no reaction was observed for high-risk mutations such as ASXL1, SRSF2, and the absence of CALR [160,161].

### 6.2. Methylation as a Prognostic Biomarker

Inhibitors of histone deacetylases (HDACi) and DNA methyltransferase enzymes (DNMTi) are included in the new therapeutic scenario. Currently in the experimental phase, imetelstat, a 13-mer lipid-conjugated oligonucleotide that uniquely targets the RNA template of human telomerase reverse transcriptase, has received interest for its potential to treat MF and essential thrombocythemia ET [162,163]. In a pilot study involving 33 patients with intermediate-2 and high-risk MF, patients harboring JAK2V617F, SF3B1, or U2AF1 mutations showed a trend of a better response to treatment, whereas those with the ASXL1 mutation, often associated with poor prognosis, showed a limited benefit. Interestingly, imetelstat also achieved a significant reduction in the mutant allele burden of JAK2, MPL, and CALR. However, the study also highlighted the challenges associated with clonal heterogeneity in MPNs. The presence of additional mutations influenced both the depth and duration of the therapeutic response [164]. Mutations with adverse prognostic implications, such as ASXL1, EZH2, and U2AF1, showed some degree of responsiveness to imetelstat, suggesting the drug’s potential to target high-risk clones. Conversely, SF3B1 and TP53 mutations persisted despite treatment, underscoring the limitations of current approaches in fully addressing clonal evolution in high-risk disease [165].

### 6.3. Epigenetic Dysregulation

Epigenetic dysregulation is important in the development of MPNs, and understanding how JAK inhibitor therapy affects the epigenetic landscape is vital to understanding its therapeutic advantages. HDACis are also being studied for MPN treatment. Vorinostat, a pan-HDAC inhibitor tested in PV and ET, demonstrated some efficacy in regulating cell proliferation, differentiation, and apoptosis in neoplastic populations. While some patients exhibited a decrease in leukocyte and platelet counts, splenomegaly, and pruritus, only minor decreases in JAK2 V617F burden were identified [166]. The effect of vorinostat on clinical trial samples was studied using an epigenetic aging signature based on DNA methylation (DNAm) of three genes (*ASPA*, *ITGA2B*, and *PDE4C*). DNAm, a major transcription regulatory mechanism, is linked to aging via the “epigenetic clock”. Diverging DNAm landscapes in older people have been correlated to neoplastic illnesses, particularly myeloid malignancies [167]. The analysis found a higher-than-expected methylation age (MA) in PV patients compared to ET patients, with greater JAK2 V617F allele load across both groups. Paired analyses indicated that after six months of vorinostat therapy, ET patients exhibited a significant normalization of MA, aligning with clinical response [58]. In contrast, non-responders showed persistently altered methylation patterns, suggesting that vorinostat failed to override the mechanisms driving these changes. The normalization of DNAm patterns appears to be associated with positive treatment outcomes, warranting further exploration of whether similar effects are observed with other MPN therapies, including JAK inhibitors [168]. Panobinostat and romidepsin, used in other cancers, have shown promise, but toxicity remains a major concern in most clinical studies. Givinostat, an HDACi with a better tolerability, demonstrated initial clinical benefits in about two-thirds of PV patients in phase I/II trials, with sustained responses exceeding 80% in long-term follow-up. These findings suggest that epigenome-targeting therapies could hold potential in MPN management [169]. Additionally, bromodomain and extra terminal (BET) inhibitors, which target epigenetic reader proteins involved in transcription and chromatin remodeling, have shown responses in mouse models of MF and are now undergoing clinical trials [170,171].

### 6.4. Role of Hypomethylating Agents

Hypomethylating agents such as azacytidine (AZA) and decitabine have shown utility in managing advanced myeloid disorders, including MPN progression to AML. AZA, a cytosine nucleotide analog, integrates into RNA, disrupting metabolism and protein synthesis. It is widely used for MDS and AML and shows some efficacy in managing MPN-AML progression. Decitabine, a deoxycytidine analog, is incorporated into DNA, binding to methyltransferases and inactivating them. At lower doses, decitabine exhibits hypomethylating activity, reactivating silenced genes [172,173]. Three protein families—PIAS, SOCS, and SHP—have been shown in studies to negatively affect JAK2/STAT signaling. In MPN patients, *SHP1* hypermethylation was found in bone marrow mononuclear cells, resulting in lower *SHP1* expression and loss of negative JAK2/STAT signaling regulation. AZA was able to restore *SHP1* expression in hypermethylated MPN cells, which inhibited JAK2/STAT activity [174]. These data indicate that restoring SHP1 expression with AZA could improve treatment outcomes in MPN patients when paired with standard therapy. As previously noted, constitutive mobilization of CD34 cells in PMF patients has been linked to proteolytic disruption of the CXCR4/SDF-1 axis and decreased CXCR4 expression. The CXCR4 promoter in JAK2V617F-positive cells and CD34 cells from PMF patients have been shown to be hypermethylated at CpG islands. AZA enhanced CXCR4 expression. This implies that AZA has a direct impact on aberrant CXCR4 methylation. Hence, it could support the actions of demethylating drugs in PMF by limiting clonal progenitor development while promoting normal progenitor re-emergence [55]. Treatment options for MPN-accelerated phase (MPN-AP) and blast phase (MPN-BP) are broadly similar, but there is currently no standard approved therapy. Most protocols include hypomethylating agents in combination with ruxolitinib. For MPN-BP patients with *IDH1* or *IDH2* mutations, targeted therapies such as ivosidenib or enasidenib have been used. In cases involving *FLT3* mutations, gilteritinib has been incorporated into the treatment regimen [175,176,177].

### 6.5. Innovations in MPN Therapy: Therapeutic Implications of DNA Methylation

The future of therapy for MPNs focus on targeted treatments that address the molecular and epigenetic mechanisms underlying the disorders. Innovations include drugs like JAK2 inhibitors, HDAC inhibitors, and lysine-specific demethylase 1 (LSD1) inhibitors, which aim to modify disease progression rather than merely controlling symptoms. These drugs explore novel pathways to restore normal hematopoietic function, also improving quality of life and survival [178]. Histone acetylation is crucial for controlling chromatin accessibility and gene expression. Increased acetylation is related to open chromatin and active transcription, whereas decreasing acetylation causes chromatin condensation and transcription inhibition. HDACs compress the DNA/histone complex, inhibiting transcription. Beyond chromatin regulation, HDACs influence apoptotic pathways by balancing pro- and anti-apoptotic proteins. As a result, HDAC inhibitors can cause cell cycle arrest, differentiation, and death, particularly in malignant cells [179]. Several HDACis have been studied as potential treatments for MPNs in both preclinical and clinical settings. Givinostat reduced the mean JAK2 V617F allele load in PV and ET patients but not in MF cases. Panobinostat suppresses downstream JAK/STAT signaling, reducing phosphorylation of STAT5, STAT3, and AKT, as well as pro-inflammatory cytokine levels (e.g., IL-6, TNFα). HDACis have been tested in combination with ruxolitinib, resulting in a decrease in spleen volume, improvement in bone marrow fibrosis, and a decrease in JAK2 V617F allele load [180]. Givinostat was approved in the USA on 21 March 2024 for the treatment of Duchenne muscular dystrophy (DMD) in patients aged 6 years and older. The GIV-IN PV study, an ongoing phase III randomized trial, is currently evaluating the efficacy and safety of the drug compared to HU in high-risk, JAK2V617F-positive PV patients. Its mechanism of action is based on the inhibition of the downstream pathways of JAK2, blocking the bone marrow stimulation. In clinical trials, it has demonstrated cytoreductive properties and the ability to counteract symptoms such as pruritus [181]. It has been tested as a single agent and in combination with HU, demonstrating good tolerability [182]. Idasanutlin and the more recent KRT232 are oral inhibitors of MDM2 that have shown a positive response in 58% of patients, with effects lasting for more than 16 months. The drugs work by targeting the P53-MDM2 pathway. In patients with the JAK2V617F mutation, MDM2—a natural suppressor of P53—is found to be overactive in hematopoietic progenitor cells, resulting in decreased levels of P53. Idasanutlin’s objective is to re-establish the proper balance in the P53-MDM2 interaction. Currently, it is being evaluated in a phase II trial for patients who are unresponsive to HU [183,184]. A clinical study is underway for IMG7289 (bomedemstat), an LSD1 inhibitor. LSD1 is a regulatory enzyme involved in hematopoiesis that is overexpressed in MPNs [185]. Its inhibition in murine models with JAK2 mutations has proven effective in reducing leukocytosis and splenomegaly [186].

However, few clinical trials implementing methylation-targeted therapies in MPNs have been organized (Table 4).

## 7. Conclusions and Future Perspectives

The role of methylome has gained a central role in MPNs pathogenesis and progression in the last years, making the relationship between epigenetic dysregulation and the development of neoplasms, including hematological ones, clear and evident. Indeed, abnormal DNA methylation patterns, such as hypermethylation of tumor suppressor genes and hypomethylation of oncogenes, are important driving factors in gene expression in these illnesses, influencing cellular proliferation, differentiation, and death.

These epigenetic modifications are further influenced by driver mutations like JAK2, CALR, and MPL, as well as chronic inflammation, resulting in a dynamic and ever-changing methylation landscape. Advances in understanding the methylome may reveal its potential as a biomarker and a therapeutic target. There are signs of distinctive methylation profiles among the subtypes of MPN, and different patterns of hyper- and hypomethylation have been associated with disease-specific pathways. Therapeutic interventions targeting the methylome, including hypomethylating agents like AZA and decitabine, have demonstrated efficacy in reactivating silenced tumor suppressor genes and normalizing aberrant methylation profiles.

Furthermore, the epigenetic effects of JAK inhibitors like ruxolitinib indicate the therapeutic value of targeting methylation in MPNs. Emerging medicines aiming at rectifying particular methylation alterations, when paired with methods to address chronic inflammation and clonal heterogeneity, show promise for changing disease trajectories and improving outcomes.

In the near future, the acquired cognitions may find clinical use by improving diagnostic and prognostic capabilities for patients with MPNs. For instance, technological advancements in cell-free DNA (cfDNA) liquid biopsy have triggered exponential growth in numerous clinical applications. Even if cfDNA-based liquid biopsy has made significant strides in personalizing cancer treatment, the exploration and translation of epigenetics in liquid biopsy to clinical practice is still nascent. Recent clinical applications of epigenetics-based cfDNA liquid biopsy revolve around DNA methylation in screening and early cancer detection, leading to the development of multi-cancer early detection tests and the capability to pinpoint tissues of origin. The clinical application of epigenetics in cfDNA liquid biopsy in minimal residual disease, in monitoring, and in surveillance are at their initial stages. However, adapting the implementation of liquid biopsy workflow worldwide and developing point-of-care testing holds great potential to improve chronic myeloproliferative neoplasms outcomes [187].

Further, the use of artificial intelligence may improve the effectiveness of data obtained from the epigenetic analysis of patients with MPNs. Machine learning (ML) is a subfield of artificial intelligence that allows a computer system to create accurate predictions and make decisions built on extrapolations based on historical data without explicit programming. ML potentially plays a role yet to be fully exploited in medicine, opening new scenarios [188,189]. In particular, in the context of MF, registry data of 1386 patients followed in 60 different Spanish centers were analyzed by this computational approach [190] to create a new score called AIPSS. The training set data modeled overall survival and leukemia-free survival based on standard clinical features collected at diagnosis, establishing an individualized prediction for every single patient. The baseline parameters considered by this model are age, gender, presence or absence of constitutional symptoms, leukoerythroblastosis, hemoglobin, number of leukocytes and platelets, and peripheral blasts.

A more accurate understanding of the disease both at the individual and population levels might be possible with extensive epigenetic screening, as early identification of MPN and adequate prognostic evaluation remain challenges for physicians. Furthermore, assessing DNA methylation gene alterations in MPN patients may help physicians to create a customized treatment plan by establishing a correlation between the symptom load and the incidence of thrombosis and probably predict the response to specific treatments [191].

Future research should also focus on the methylome as a diagnostic and predictive tool, while improving the development of methylation-targeted medicines.

## Figures and Tables

**Table 1:** Summary of mutations involved in methylome changes.

Mutation	Type	Effect	Consequence
TET2	Loss-of-function	Impaired DNA demethylation	Clonal hematopoiesis, increased risk of transformation to leukemia
DNMT3A	Loss-of-function	Altered DNA methylation	Disrupted hematopoietic differentiation
EZH2	Loss-of-function	Reduced H3K27 methylation	Deregulated gene silencing, enhanced stem cell renewal
ASXL1	Loss-of-function	Dysregulation of PRC2 activity	Altered chromatin structure, defective hematopoiesis
IKZF1	Deletion	Loss of myeloid gene repression	Increased risk of leukemic transformation
IDH1/IDH2	Gain-of-function	Altered α-KG-dependent enzymes	Disrupted DNA and histone methylation, leukemic progression

**Table 2:** Influence of methylation pattern in MPNs.

Methylation Pattern	Clinical Effects in MPNs
Hypermethylation in PV and ET	Suppresses genes involved in hematopoiesis and inflammation
Hypomethylation in PMF	Leads to aberrant activation of oncogenes, contributing to hematopoietic dysregulation
Altered Methylation of miRNAs	Associated with increased cell proliferation and leukemic transformation
ASXL1 Hypermethylation	Promotes clonal evolution and leukemic transformation
TET2 Hypomethylation	Disrupts DNA hydroxymethylation, increasing risk of clonal hematopoiesis
Hypermethylation of CXCR4	Reduces hematopoietic stem cell retention in bone marrow, leading to excessive mobilization in PMF
Hypermethylation due to Chronic Inflammation	Enhances genomic instability and drives disease progression, especially in MF

**Table 3:** Mechanisms, epigenetic effects, and corresponding observed clinical responses of different pharmacologic agents.

Pharmacology Agent	Mechanism of Action	Epigenetic Effects	Clinical Impact
Hydroxycarbamide	Inhibits ribonucleotide reductase, blocking DNA synthesis	Alters DNA methylation, restores SPI1 and RUNX1 expression	First-line treatment for MPNs, reduces leukocyte/platelet counts
Ruxolitinib	JAK1/2 inhibitor, reduces cytokine signaling	Alters DNA methylation, histone modifications (H3K9 methylation)	Reduces spleen size, constitutional symptoms in MF
Fedratinib	Selective JAK2 inhibitor, also inhibits FLT3	Inhibits BRD4 (BET protein family), affects chromatin remodeling	Used for MF, reduces inflammatory cytokine production
Momelotinib	JAK1/2 inhibitor with additional activity on ACVR1	Limited data on epigenetic effects	Effective in MF patients with anemia, improves transfusion independence
Givinostat	HDAC inhibitor, blocks JAK2 downstream signaling	Alters histone acetylation, affects JAK/STAT pathway	Shows cytoreductive effects in PV, being evaluated for high-risk cases
Vorinostat	Pan-HDAC inhibitor, affects cell proliferation and differentiation	Alters DNA methylation, linked to epigenetic age reversal	Used in PV and ET, limited effect on JAK2 V617F burden
Panobinostat	HDAC inhibitor, suppresses JAK/STAT signaling	Reduces phosphorylation of STAT5, STAT3, AKT	Used in MF, reduces spleen volume and inflammation
Decitabine	DNMT inhibitor, reactivates silenced genes	Restores SHP1 expression, affects CXCR4 methylation	Used in MPN-AML progression, in combination with JAK inhibitors
Azacitidine	Hypomethylating agent, integrates into RNA	Reactivates silenced tumor suppressor genes	Used in advanced myeloid disorders, can restore normal progenitor growth
Imetelstat	Telomerase inhibitor, blocks RNA template	Reduces JAK2, MPL, and CALR mutant allele burden	Shows promise in MF and ET, affects high-risk clonal populations

**Table 4:** Clinical trials implementing methylation-targeted therapies in MPNs. https://clinicaltrials.gov (accessed on 19 March 2025).

Study Title	Number	Status	Conditions	Interventions	Sponsor	Study Type
Impact of Epigenetic Age on Clinic-biological Presentation and Prognosis in Myeloproliferative Neoplasms Epigenetic Age in Myeloproliferative Neoplasms (EpiC)	22,328	Recruiting	Myeloproliferative neoplasms	Biological: Assessment of the epigenetic age	University Hospital Bordeaux	Observational
Curcumin to Improve Inflammation and Symptoms in Patients With Clonal Cytopenia of Undetermined Significance, Low Risk Myelodysplastic Syndrome, and Myeloproliferative Neoplasms	63,486	Recruiting	Clonal Cytopenia of Undetermined Significance Essential Thrombocythemia Myelodysplastic Syndrome	Procedure: Biospecimen Collection Procedure: Bone Marrow Aspiration Procedure: Bone Marrow Biopsy	University of Southern California	Interventional
